# Selective Removal of Plasma Proteins by Double-Filtration Plasmapheresis in Canine Blood: An Ex Vivo Study and Retrospective Report of In Vivo Clinical Treatments in Three Dogs

**DOI:** 10.3390/vetsci12060528

**Published:** 2025-05-29

**Authors:** Roberta Troia, Claudia Iannucci, Lisa Niemann, Alessio Vigani

**Affiliations:** Section of Small Animal Emergency and Critical Care, Department of Small Animals, Vetsuisse Faculty, University of Zurich, 8057 Zurich, Switzerland; roberta.troia@uzh.ch (R.T.); claudia.iannucci@uzh.ch (C.I.); lisa.niemann@uzh.ch (L.N.)

**Keywords:** INUSpheresis^®^, extracorporeal apheresis, immunoglobulin, double-filtration plasmapheresis, dog

## Abstract

Double-filtration plasmapheresis (DFPP) is a plasma-exchange modality that allows selective clearance of high-molecular-weight proteins, potentially minimizing albumin loss and the need for volume replacement. Reports concerning DFPP use in dogs are scarce. This study evaluates the quantitative net loss of different plasma proteins fractions in an ex vivo model using canine blood processed via DFPP. DFPP treatment with INUSpheresis^®^ processing 1.5, 2, and 3 plasma volumes (PVs) was performed. Plasma proteins fractions were measured in the reservoir blood bag at baseline (pre-treatment) and in the effluent bag at the end of each target PV exchanged to calculate the net loss of selected plasma proteins. At 1.5 PV, net globulin and albumin loss was 41 and 25% respectively. At 3 PV, net globulin and albumin loss was 47 and 40%, respectively. Fibrinogen concentration was unmeasurable low in the reservoir blood bag after processing 1.5 PV. INUSpheresis^®^ allows selective plasma proteins removal, with a sparing effect on albumin at 1.5 PV. Selectivity is, however, progressively reduced with incremental target PV. A description of five DFPP treatments in three dogs is additionally presented. Semi-selective protein removal was also demonstrated in vivo, with a significantly lower percentage reduction in albumin compared to total globulin (*p* = 0.01) and fibrinogen (*p* = 0.007).

## Simple Summary

Double-filtration plasmapheresis is a semi-selective plasma exchange modality that removes high-molecular weight substances, including immunoglobulin and immune complexes, while minimizing the loss of albumin and the consequent need for substitution fluids. In contrast to conventional plasmapheresis, with double-filtration plasmapheresis the volume of discarded plasma is significantly reduced, lowering the volume of the replacement fluid and the associated risk for transfusion reactions. This technique has been increasingly used in human medicine to treat different immune-mediated, hematological, and neurological conditions, but reports regarding its use in dogs are scarce. The aim of this study is to evaluate the quantitative net loss of different plasma proteins fractions in an ex vivo model using canine blood with double-filtration plasmapheresis. A semi-selective protein removal is demonstrated ex vivo, with a significant sparing effect on albumin when 1.5 plasma volume are processed. A description of the use of double-filtration plasmapheresis in three dogs affected by immune-mediated diseases is additionally presented. The obtained results in vivo demonstrate that a semi-selective protein removal occurs in dogs, as the median percentage reduction in albumin is significantly lower compared to total globulin and fibrinogen. Double-filtration plasmapheresis seems to be a promising plasma-exchange modality deserving investigations in veterinary patients.

## 1. Introduction

Double-filtration plasmapheresis (DFPP) is a semi-selective plasma-exchange modality that removes high-molecular-weight substances including immunoglobulin, immune complexes, and toxins, potentially minimizing the loss of albumin and the consequent need for substitution [1,2,3,4]. In DFPP, the plasma separator is responsible for extracting plasma across the membrane. The second filter is a plasma fractionator, that depending on the pore sizes of the membrane, allows the selective removal of plasma proteins based on their molecular weight. While the large-molecular-weight components are discarded, small-molecular-weight proteins including albumin are returned to the patient. In contrast to conventional plasma exchange, the volume of discarded plasma is significantly lower, hence reducing the overall volumes of the replacement fluids required and the risks associated with heterologous plasma transfusions [4].

Due to the efficient removal of immunoglobulins and immune complexes, a large number of immune-mediated diseases in human medicine can be treated through DFPP, including myasthenia gravis, chronic inflammatory demyelinating polyneuropathy, polymyositis, lupus nephritis, rheumatoid arthritis, the prevention of antibody-dependent xenograft rejection, and many other conditions [4,5,6]. In addition to this, its application in human medicine is also reported in diseases of metabolic origin, such as familial hypercholesterolemia or lipoprotein hyperlipoproteinemia, reducing the risk for cardiovascular events and pro-inflammatory processes [6]. The removal of both coagulation and anticoagulation factors with a large molecular weight is also expected, requiring coagulation monitoring for the risk of clinical bleeding [3,7].

The use of DFPP has been scarcely documented in dogs. Lippi et al. described the treatment of a dog with multiple myeloma and hyperviscosity syndrome with three sessions of DFPP. A significant reduction in serum total proteins and globulins was found following each treatment, allowing for a complete, although temporary clinical resolution of the hyperviscosity syndrome [1]. Similarly, the use of DFPP has been reported to successfully treat severe hyperproteinemia and hyperglobulinemia secondary to *Leishmania infantum* in three dogs [2]. No complications were reported. Despite the occurrence of mild-to-moderate hypoalbuminemia, none of the dogs required exogenous plasma or albumin supplementation after the procedure [1,2]

INUSpheresis^®^ (INUS Medical Devices AG, Steinhausen, Switzerland) is a specific type of DFPP platform developed for use in humans, that has shown therapeutic potential for the treatment of several metabolic and non-metabolic diseases, neuropathies, as well as for the removal of protein-bound environmental toxins. The pore size of the membrane of the plasma fractionator TKM58^®^ (INUS Medical Devices AG, Steinhausen, Switzerland) showed a significant reduction in γ-globulin levels in human blood [6].

The aim of the present study is to evaluate ex vivo the selective net loss of different plasma proteins in canine blood by DFPP treatment with INUSpheresis^®^ and TKM58^®^ processing from 1.5 up to 3 plasma volumes. The net percentage reduction in different plasma proteins was additionally assessed and compared evaluating in vivo clinical treatments in three dogs.

## 2. Materials and Methods

### 2.1. Ex Vivo Study

**Figure 1:**
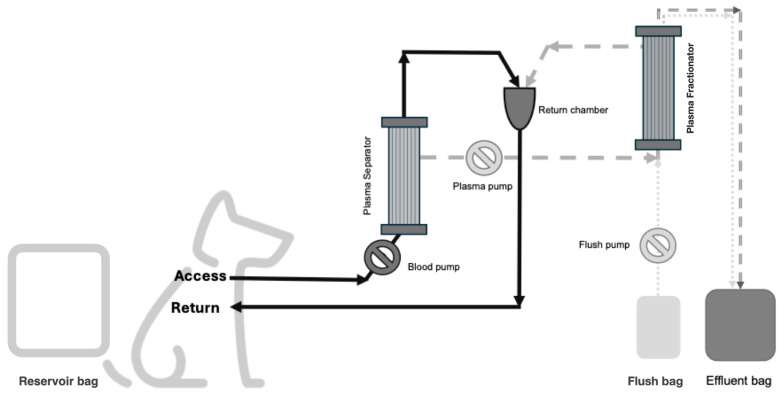
The extracorporeal circuit of double-filtration plasmapheresis. The continuous black line identifies the blood path from the patient/reservoir bag (access), into the plasma separator, and back to the patient/reservoir bag (return). The dashed grey line identifies the plasma pathway from the plasma separator, into the plasma fractionator, and back to the return chamber or the effluent bag. The dotted grey line represents the flush path from the flush bag, into the plasma fractionator, to the effluent bag. The “reservoir bag” represents the “patient” in the ex vivo study. The effluent bag collects the plasma proteins removed by the plasma fractionator and the volume of the automated flushes. The flush bag is represented by the sterile saline solution used for the automated flushes of the plasma fractionator during the session.

This study conducted at the University Veterinary Hospital of XXX assessed the effect of DFPP treatment with INUSpheresis^®^ on canine blood. Canine blood (total volume 1683 mL) was obtained mixing in a single reservoir bag, four units of packed red blood cells (803 mL total, D.E.A. negative, anticoagulant CPDA), and four units of fresh frozen plasma (880 mL total). Packed red blood cells and fresh frozen plasma had been purchased from a commercial veterinary blood bank (Banco Sangue Animal, Rua de João de Deus, nº 741, 4100-462–Porto, Portugal). Non-fractionated heparin (25000 units; Heparin sodium 5000 I.U./mL) was added to the solution to avoid any risk of clot formation within the reservoir. The commercially available extracorporeal circuit for INUSpheresis^®^ with a standard plasma separator and a TKM58^®^ as plasma fractionator were used for this study. Arterial and venous lines were connected to the reservoir bag. Normal saline 0.9% was used as the circuit priming solution. The priming volume of the circuit was 250 mL. A continuous DFPP treatment was performed processing incremental volume, respectively, 1.5, 2, and 3 target plasma volumes (PVs) over 180 min. Blood flow was set at 100 mL/min. Plasma filtration fraction across plasma separator was set fixed at 30%. Automatic flushing of the plasma fractionator with 150 mL of normal saline 0.9% were performed at each target PV time point to allow collection of the removed proteins and to eliminate possible fibrin clots within the plasma fractionator. The volume of each flush was accounted as part of the overall effluent volume (Figure 1).

Our reference values were the ones referred to the reservoir bag at the beginning of the session (baseline, pre-treatment). Blood and plasma samples were collected from the reservoir bag at baseline (pre-treatment), and serially from the effluent bag at each target PV exchange: 1.5, 2, and 3 PV. The samples were processed as follows: heparin whole blood was used to measure hematocrit and total proteins using an in-house centrifuge and refractometer. Plasma was obtained after centrifugation (1000 g × 10 min) and stored at −30 °C until further analyses. All the collected samples from the reservoir bag and the effluent bag were used for the following evaluations: total proteins using the Biuret assay (Cobas C 501, Roche Diagnostics, Basel, Switzerland); plasma fibrinogen concentrations using the Clauss method (Start Max, Stago, Switzerland); and electrophoresis in agarose gel for the measurement of albumin, α1-globulin, α2-globulin, β1-globulin, β2-globulin, and γ-globulin concentrations (Hydrasys 2 Scan Focusing, Sebia Swiss Gmbh, Wollerau, Switzerland). All the analyses were performed at the Vetsuisse Central Veterinary Laboratory.

The mass in grams (g) of the plasma protein fractions at baseline was calculated as follows: [(solute concentration in reservoir bag (g/L) × total volume (L) reservoir bag)]. The mass in grams (g) of the plasma protein fractions being removed at 1.5, 2, and 3 processed PV was calculated as follows: [(solute concentration in effluent bag (g/L) × total volume (L) effluent bag)].

The net percentage loss of the different protein fractions was calculated as follows: [(Mass (g) of solute in the effluent bag at target PV/(Mass (g) of solute in reservoir bag at baseline)] × 100.

### 2.2. Clinical Cases

We evaluated also retrospectively the results of three clinical cases, corresponding to a total of five DFPP treatment sessions performed between June 2024 and January 2025. For each patient, blood and plasma volumes were calculated as follows:

Blood volume (mL) = body weight kg × 90

Plasma volume (mL) = blood volume × (1 − hematocrit value)

Prior to the first DFPP treatment, each dog was sedated for the placement of a silicon double-lumen hemodyalisis catheter (Hemo-Cath 11.5 F, 20 cm; MedComp) in the right external jugular vein. No sedation or anesthesia was required for any of the dogs during the DFPP treatments. Systemic anticoagulation during each session was maintained with intravenous unfractionated heparin targeting an activated partial thromboplastin time (aPTT) between 180–220 s. The aPTT measurements were repeated at intervals of 30 min during each DFPP session. For each patient, pre-treatment and post-treatment concentrations of total proteins, albumin, total globulin, α1-globulin, α2-globulin, β1-globulin, β2-globulin, γ-globulin, and fibrinogen were measured during all the treatments. Samples were also collected from the effluent bag as part of standard clinical protocol at the end of each treatment.

The net percentage protein reduction was calculated using the following formula: (pre-treatment solute concentration–post-treatment solute concentration)/pre-treatment solute concentration.

The net albumin and total globulin loss expressed in g/kg was calculated as follows: (solute concentration effluent bag × total volume ml effluent bag)/patient body weight kg.

### 2.3. Statistics

Descriptive statistics were calculated and normality was assessed with the Shapiro–Wilk test. As the majority of the data were non-normally distributed, all data were expressed as the median (min–max). Non-parametric statistics (Mann–Whitney-U test and Kruskal–Wallis test with Dunn’s correction for multiple comparisons) were used to compare the net percentage reduction in different plasma proteins. The statistical analyses were performed with commercial software (Prism 10.4.1, GraphPad). Alpha was set a *p* < 0.05.

## 3. Results

### 3.1. Ex Vivo Study

Table 1, Table 2 and Table 3 summarize the laboratory data measured in the reservoir bag (baseline), as well as those obtained from the effluent bag at 1.5, 2, and 3 PV.

The baseline total plasma proteins in the reservoir bag were 63,9 g. The effluent bag had 19.7 g of total proteins removed at 1.5 PV, 25 g removed at 2 PV, and 27 g removed at 3 PV. This was equivalent to a net total plasma protein loss percentage of 32% at 1.5 PV, 40% at 2 PV, and 42% at 3 PV.

The mass of albumin in the reservoir bag was 39 g at baseline. The effluent bag had 9.7 g of albumin removed at 1.5 PV, 13.6 g removed at 2 PV, and 15.5 g removed at 3

PV. This was equivalent to a net albumin loss percentage of 25% at 1.5 PV, 35% at 2 PV, and 40% at 3 PV.

The mass of total globulin in the reservoir bag was 24.4 g. The effluent bag had 10 g removed at 1.5 PV, and 11.3 g removed at both 2 PV and 3 PV. This corresponded to a net total globulin loss percentage of 41% at 1.5 PV, 47% at 2 PV, and 47% at 3 PV.

The initial mass of γ-globulin in the reservoir bag was 3.2 g. The effluent bag had 1.6 g removed at 1.5 PV, and 1.8 g removed at both 2 PV and 3 PV. This corresponded to a net γ-globulin loss percentage of 50% at 1.5 PV, 57% at 2 PV, and 57% at 3 PV.

The baseline fibrinogen mass in the reservoir bag was 2.2 g. The effluent bag had 0.27 g removed at 1.5 PV, 0.61 g removed at 2 PV, and 0.61 g removed at 3 PV. The net plasma fibrinogen loss percentage was 13% at 1.5 PV, 28% at 2 PV, and 28% at 3 PV. Notably, the fibrinogen concentration in the reservoir bag became unmeasurably low (<0.4 g/L) already after processing 1.5 PV.

### 3.2. In Vivo Clinical Treatments

Case 1: A 24.5 kg male intact dog referred to the University Veterinary Hospital XXX for acute immune-mediated hemolytic anemia and glomerulonephritis underwent DFPP with INUSpheresis^®^. The estimated blood and plasma volumes were 2205 mL and 1725 mL, respectively. Protein percentage reduction was calculated, as previously stated.

A continuous DFPP treatment with INUSpheresis^®^ and TKM58^®^ as plasma fractionator was performed processing 1.7 PV (3000 mL) over 120 min. The circuit was primed with packed red blood cells and normal saline. Blood flow was set between 50−110 mL/min. Plasma filtration fraction across plasma separator was set fixed at 30%. Five automatic flushes of the plasma fractionator with 150 mL each of normal saline 0.9% were performed during the session. The net plasma volume removed was 220 mL. The replacement was performed with 220 mL of fresh frozen plasma during the treatment. The dog received additionally a unit of 220 mL of packed red blood cells during the session. Lactated Ringer was used as a rinse-back at the end of the session.

The pre-treatment hematocrit value was 12%, and post-treatment hematocrit value was 16%. The pre-treatment protein values were as follows: total protein 33 g/L, albumin 20.4 g/L, total globulin 12.6 g/L, γ-globulin 2 g/L, and fibrinogen 1.1 g/L. The plasma protein concentrations post-treatment were the following: total protein 15 g/L, albumin 9.8 g/L, total globulin 5.2 g/L, γ-globulin 0.6 g/L, and fibrinogen 0.27 g/L. The net percentage reduction was 54% for total proteins, 52% for albumin, 59% for total globulins, 70% for γ-globulins, and 75% for fibrinogen, respectively. The net albumin and total globulin loss was 1.2 g/kg and 0.8 g/kg, respectively.

No complications were encountered during the treatment. The dog underwent two additional DFPP sessions at 24 and 48 h. A final diagnosis of acute myeloid leukemia and hemophagocytic syndrome was reached after 5 days of hospital stay, and the dog was euthanized due to worsening medical conditions.

Case 2: A 45.5 kg male intact dog referred to the University Veterinary Hospital XXX for acute azotemia and glomerulonephritis underwent DFPP with INUSpheresis^®^. The estimated blood and plasma volumes were 4095 mL and 2839 mL, respectively. A continuous DFPP treatment with INUSpheresis^®^ and TKM58^®^ as plasma fractionator was performed processing 1.5 PV (4200 mL) over 120 min. The net plasma volume removed was 600 mL. The replacement was performed with 700 mL of fresh frozen plasma during the treatment. Lactated Ringer was used as a rinse-back at the end of the session.

The pre- and post-treatment hematocrit values were 22 and 26%, respectively. The pre-treatment protein values were as follows: total protein 50.8 g/L, albumin 19.8 g/L, total globulin 31.0 g/L, γ-globulin 2.2 g/L, and fibrinogen 6.1 g/L. The plasma protein concentrations post-treatment were the following: total protein 31.0 g/L, albumin 16.8 g/L, total globulin 14.8 g/L, γ-globulin 1.6 g/L, and fibrinogen 1.5 g/L. The net percentage reduction was 38% for total proteins, 15% for albumin, 52% for total globulins, 27% for γ-globulins, and 75% for fibrinogen, respectively. The net albumin and total globulin loss was 0.9 g/kg and 2 g/kg, respectively.

At 24 h, a second DFPP treatment processing 1.5 PV was again performed. The net plasma volume removed was 120 mL. The replacement was performed with 220 mL of fresh frozen plasma during the treatment. Lactated Ringer was used as a rinse-back at the end of the session. The pre- and post-treatment hematocrit values were 26 and 27%, respectively. The pre-treatment protein values were as follows: total protein 44 g/L, albumin 20.9 g/L, total globulin 23.2. g/L, γ-globulin 1.9 g/L, and fibrinogen 4.6 g/L. The plasma protein concentrations post-treatment were the following: total protein 23.4 g/L, albumin 14.1 g/L, total globulin 9.3 g/L, γ-globulin 0.8 g/L, and fibrinogen 0.6 g/L. The net percentage reduction was 47% for total proteins, 32% for albumin, 60% for total globulins, 58% for γ-globulins, and 87% for fibrinogen, respectively. The net albumin and total globulin loss was 0.6 g/kg and 0.9 g/kg, respectively.

No complication occurred during the treatments. The dog underwent an additional DFPP treatment and a total of 7 dialysis treatments during hospitalization. There was no improvement in azotemia, and after 18 days of hospital stay, the owners elected euthanasia.

Case 3: A 19.7 kg male intact dog referred to the University Veterinary Hospital XXX for acute immune-mediated hemolytic anemia underwent DFPP with INUSpheresis^®^. The estimated blood and plasma volumes were 1773 mL and 1008 mL, respectively. A continuous DFPP treatment with INUSpheresis^®^ and TKM58^®^ as plasma fractionator was performed processing 2 PV (2100 mL) over 120 min. Whole blood was used for circuit priming. The net plasma volume removed was 50 mL. The replacement was performed with 220 mL of fresh frozen plasma during the treatment. Lactated Ringer was used as a rinse-back at the end of the session.

The pre- and post-treatment hematocrit values were 20 and 29%, respectively. The pre-treatment protein values were as follows: total protein 36 g/L, albumin 18.2 g/L, total globulin 17.8. g/L, γ-globulin 1.9 g/L, and fibrinogen 3.1 g/L. The plasma protein concentrations post-treatment were the following: total protein 19 g/L, albumin 11.2 g/L, total globulin 7.8 g/L, γ-globulin 1.2 g/L, and fibrinogen 0.4 g/L. The net percentage reduction was 47% for total proteins, 38% for albumin, 56% for total globulins, 37% for γ-globulins, and 87% for fibrinogen, respectively. The net albumin and total globulin loss was 0.5 g/kg and 0.77 g/kg, respectively.

At 24 h, a second DFPP treatment processing 1.5 PV was again performed. The net plasma volume removed was 70 mL. The replacement was performed with 150 mL of fresh frozen plasma during the treatment. Lactated Ringer was used as a rinse-back at the end of the session. The pre- and post-treatment hematocrit values were 36 and 30%, respectively. The pretreatment protein values were as follows: total protein 29 g/L, albumin 15.7 g/L, total globulin 13.3. g/L, γ-globulin 1.7 g/L, and fibrinogen 3 g/L. The plasma protein concentrations post-treatment were the following: total protein 16 g/L, albumin 10 g/L, total globulin 5.9 g/L, γ-globulin 0.6 g/L, and fibrinogen 0.4 g/L. The net percentage reduction was 45% for total proteins, 36% for albumin, 64% for total globulins, 55% for γ-globulins, and 87% for fibrinogen, respectively. The net albumin and total globulin loss was 0.96 g/kg and 1 g/kg, respectively.

No complication occurred during the treatments. There was a significant improvement in the general clinical condition and no evidence of persistent hemolysis after the two DFPP treatments. The dog was discharged at day 6 with immune-suppressive treatment. At the following re-checks performed, the dog’s clinical status was brilliant, and there was no sign of disease recurrence.

**Figure 2:**
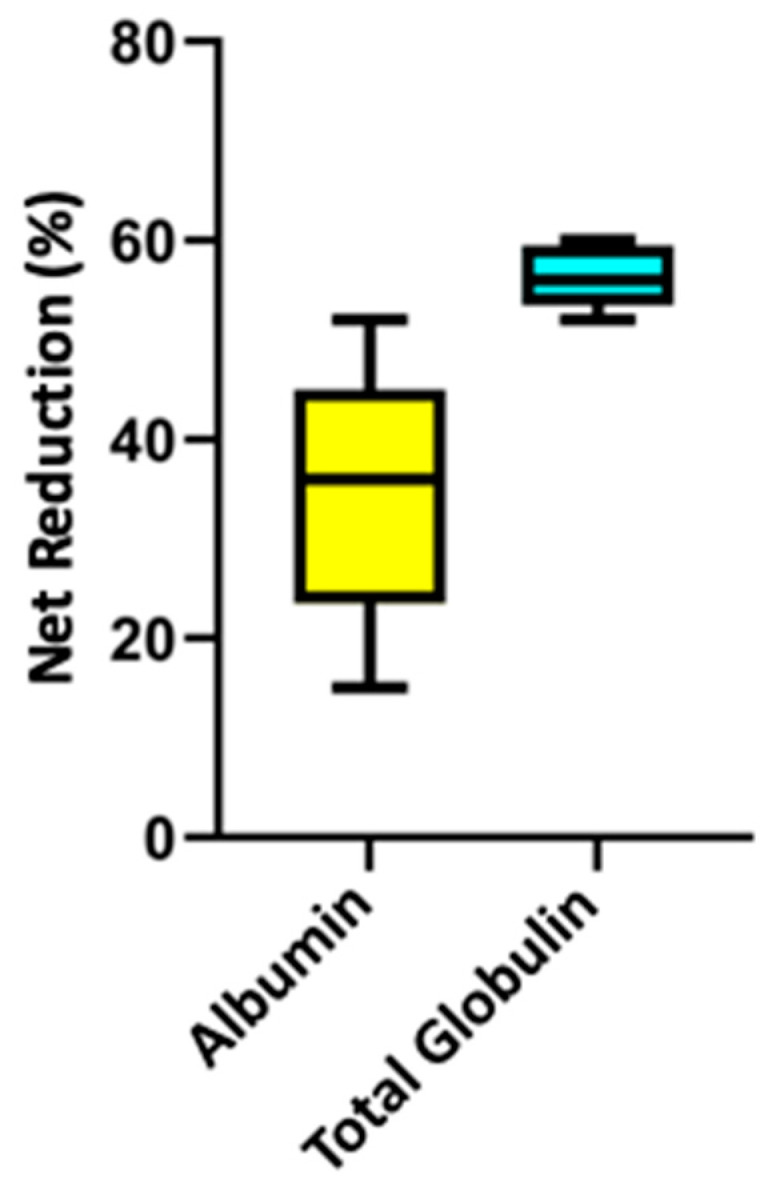
Box and whisker plot comparison of the median net percentage protein reduction in albumin vs. total globulin from five double-filtration plasmapheresis sessions in three dogs. The central line represents the median, the boxes represent the interquartile range, and the whiskers represent the minimum and maximum values. Albumin net percentage reduction resulted significantly lower compared to globulin net percentage reduction (*p* = 0.01) by Mann–Whitney U test.

**Figure 3:**
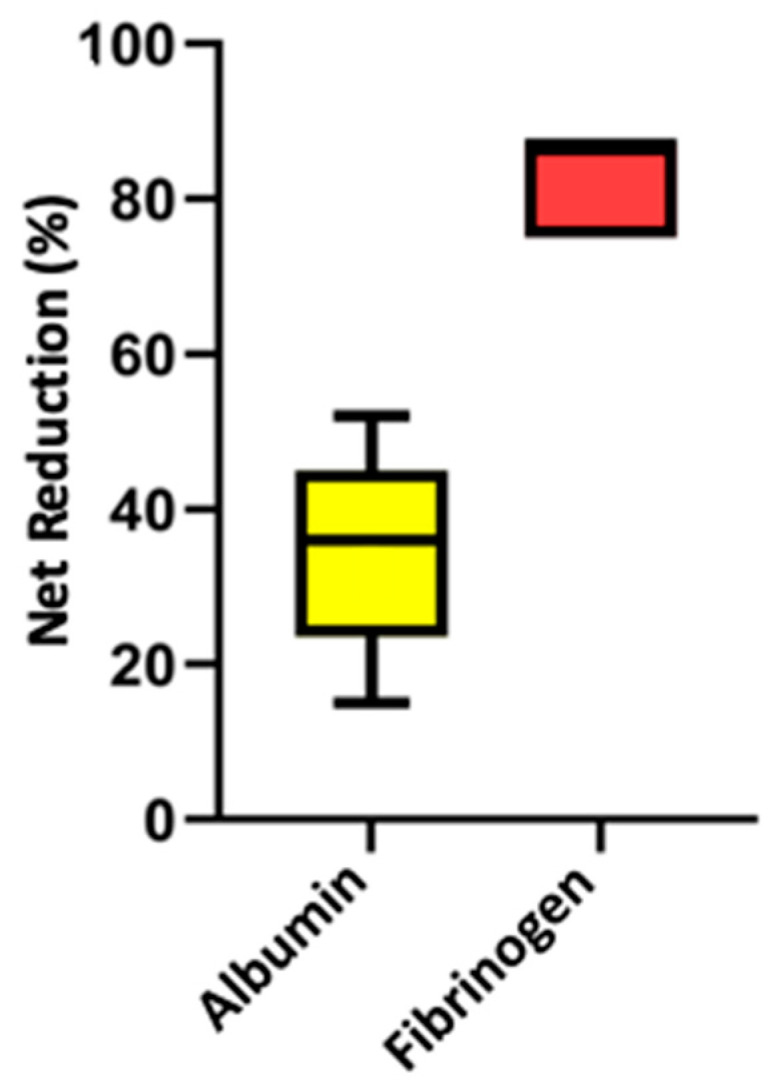
Box and whisker plot comparison of the median net percentage protein reduction in albumin vs. fibrinogen from five double-filtration plasmapheresis sessions in three dogs. The central line represents the median, the boxes represent the interquartile range, and the whiskers represent the minimum and maximum values. Albumin net percentage reduction resulted significantly lower compared to fibrinogen net percentage reduction (*p* = 0.01) by Mann–Whitney U test.

The median processed PV in the five treatment sessions described was 1.6. The median values and the ranges for the net percentage proteins reductions documented for the reported five in vivo clinical treatments were the following: 36% (15−52) albumin; 56% (52−60) total globulin; 58% (27−70) γ-globulin; and 87% (75−87) fibrinogen. The net percentage reduction in albumin resulted significantly lower than the one documented for total globulin (*p* = 0.01) and for fibrinogen (*p* = 0.007) (Figure 2 and Figure 3). No difference was detected in the comparison between the net percentage reduction in total globulin vs. γ-globulin (*p* = 0.9). No difference was detected between the net percentage reduction in albumin vs. γ-globulin (*p* = 0.2).

The median values for the net amount of albumin and globulin loss expressed in g/kg were 0.9 g/kg (0.5−1.2) and 0.9 g/kg (0.8−2), respectively.

## 4. Discussion

Double-filtration plasmapheresis is a recently developed apheretic technique used in human medicine to treat several pathological conditions [1,2,3,4]. The veterinary experience concerning its use is still scarce and limited to few case reports [1,2]. Moreover, there are still knowledge gaps regarding the efficiency of removal of the different plasma components, as well as regarding optimal DFPP protocols in human and veterinary patients [1,2,4,8,9].

Our study aimed at measuring the ex vivo net loss percentage of different plasma proteins in canine blood by continuous DFPP treatment with INUSpheresis^®^ with a TKM58^®^ as plasma fractionator, processing from 1.5 up to 3 plasma volumes.

The results of this study highlight that the removal of plasma proteins by INUSpheresis^®^ is semi-selective, showing a significant sparing effect on albumin, compared to conventional therapeutic plasma exchange at 1.5 PV. Moreover, protein removal seems to be based not only on molecular size, but also on additional factors including adsorption within the filter and dilution by automated flushes.

DFPP should minimize albumin loss and the subsequent need for substitution fluids, due to the small molecular weight of this protein. Our results show good albumin sparing effect at 1.5 target PV, since for a 50% γ-globulin reduction, the documented net albumin loss was only 25%. Such selective protein removal at 1.5 PV, which represents a conventional target volume of exchange for clinical use of DFPP, highlights the advantage of DFPP compared to conventional therapeutic plasma exchange, where unselective protein removal occurs and the whole volume of plasma extracted has to be fully replaced [4,10]. The albumin sparing reduces substantially the requirement for replacement solutions, minimizing costs and the risks for plasma-transfusion-related complications [4,8,10].

Nonetheless, such selectivity in plasma protein removal decreased with incremental target PV, as net albumin loss reached 40% at 3 PV. The automated saline flushes performed at each target PV time point might have been responsible for the progressive albumin loss alongside incremental PV exchanged, as the volume of the plasma fractionator was removed with each flush instead of being returned to the patient.

Globulin removal was efficiently performed: total globulins and γ-globulins net loss percentage was 41 and 50% at 1.5 target PV, respectively. Globulins are large-size proteins that are directly cleared by the plasma fractionator and subsequently discarded. In humans, a filter-dependent effect on immunoglobulin clearance and protein patterns has been demonstrated following DFPP, with immunoglobulin reduction by 50% using INUSpheresis^®^ with TKM58^®^ [6], similarly to our results. Nonetheless, a decrease in globulin excretion ratio was observed with incremental target PV exchanged, with net γ-globulin removal raising only from 50% at 1.5 PV to 57% at both 2 and 3 target PV. The observed reduction in globulin clearance with increasing PV exchanges can be easily justified with membrane clogging lowering plasma fractionator efficiency. Hence, given the progressive loss of membrane selectivity and reduced globulin clearance with increasing PV exchanges, the use of >1.5 target PV does not seem clinically recommended.

A finding of notice was in regards to fibrinogen concentration. Fibrinogen is a large-size intravascular protein that is expected to be extensively cleared during DFPP treatment [3,7]. Post-treatment ex vivo fibrinogen concentration was unmeasurably low (<0.4 g/L) already after 1.5 PV exchanged (Table 1). Interestingly, based on the amount of fibrinogen detected in the effluent bag, the net loss fibrinogen percentage directly related to filtration and effluent removal ranged only between 13 to 28% from 1.5 to 3 PV. This highlights the potential role for additional mechanisms contributing to fibrinogen loss other than selected size-based protein removal in the plasma fractionator. In this regard, it is possible that part of the detected fibrinogen loss was due to its adsorption within the plasma fractionator. This seems in accordance with the role of fibrinogen as being directly involved into filter clogging with fibrin clots. Fibrinogen removal after DFPP might positively affect hemostasis in specific conditions where hyperfibrinogenemia and thrombinemia pose patients at risk for hypercoagulation and thrombotic events (e.g., nephrotic syndrome) [11]. Nonetheless, coagulation parameters and transfusion requirements should be closely monitored after apheresis, especially in patients at higher bleeding-risk or if invasive procedures are planned [12]. Previous authors have speculated additional clearance factors in DFPP independent of the protein size alone [9].

The circuit used for INUSpheresis^®^ has a volume of 250 mL. The addition of such volume to the plasma component of the reservoir bag (880 mL) used for the ex vivo study caused roughly 30% dilutional effect on plasma proteins concentration after treatment.

There are some limitations to be acknowledged when interpreting the results of our study. This was an ex vivo study; hence, the generalization of the clinical results is possible only to a limited extent. No proteomic analysis of protein absorption by the plasma fractionator membrane was performed to characterize those more subjected to potential filter adsorption. Moreover, this study relied on a mono-compartment model, hence, the role of physiologic proteins redistribution occurring during DFPP treatment in vivo cannot be assessed. However, considering that normal plasma proteins redistribution is a slow process occurring at a rate of 1−3%/h [12,13], our ex vivo results are likely mirroring those in vivo.

The primary aim of this study was to evaluate the selectivity of the plasma fractionator TKM58^®^ at incremental PV exchanged ex vivo and compare it with in vivo findings from clinical cases treated with DFFP. Duplicate analyses were not deemed necessary for the ex vivo session, although they could have potentially increased the precision of our results. Finally, the clinical implications of the present results are difficult to predict. For example, although the documented net loss of fibrinogen was significant already after 1.5 PV exchanged, the risk for clinical bleeding or need for blood products transfusions has to be assessed in clinical studies. Similarly, the actual sparing effect of DFPP on the need for plasma substitution in the clinical setting cannot be tested.

Based on the clinical treatments results presented here, DFPP with TKM58^®^ enabled to obtain a differential semi-selective plasma proteins removal also in vivo. After a median of 1.6 PV processed in the five DFPP sessions described, the median albumin reduction was 36%, resulting significantly lower from the median reduction of total globulin (56%) and the one documented for fibrinogen (87%). The albumin sparing effect was consistent in all clinical treatments, resembling the data previously reported in similar clinical case series [1,2]. The total albumin loss normalized to body weight averaged 0.9 g/kg, still highlighting the need of possible replacement in case of pre-existing hypoalbuminemia. In this regard, measuring albumin concentration in the effluent bag alongside the discarded plasma volume can be easily used to estimate the amount of post-DFPP albumin replacement. Fibrinogen removal was high also in vivo, requiring the close monitoring of coagulation and bleeding risk in the immediate post-treatment period. Interestingly, in the five DFPP sessions analyzed, no difference was documented between total globulin vs. γ-globulin percentage reduction. This might have been due to the low number of treatments analyzed. It might be also possible that globulin removal by TKM58^®^ in canine blood is not selective for the different globulin fraction.

The retrospective nature of our clinical analysis prevented the standardization of the DFPP procedure. In this regard, the plasma volume processed, as well as the volume of discarded plasma were variable, and mainly affected by the number of automated flushes imposed by the machine. This can also have affected the amount of protein recovery in the effluent after treatments. The replacement volume varied based on hemodynamic stability and bleeding tendencies of enrolled patients. Finally, the number of described sessions is low to allow meaningful statistical analysis and to reach definitive clinical conclusions.

In conclusion, INUSpheresis^®^ allows an effective and selective plasma protein removal in canine blood. Particularly at a 1.5 target PV, large-size proteins are efficiently removed through selective plasma filtration, while albumin loss is reduced compared to conventional therapeutic plasma exchange. Both the selectivity and efficiency of globulin removal are progressively reduced with increasing plasma volumes exchanged, pointing out the clinical advantage of DFPP mainly when conventional target PVs are processed. Other mechanisms including adsorption within the filter and circuit dilution play an additional role in further net protein loss after treatments. DFPP seems to promote a semi-selective protein removal also in vivo in dogs. The sparing effect of DFPP compared to standard TPE on volume replacement, treatment cost effectiveness, as well as the clinical consequences of fibrinogen loss in dogs have to be addressed in future clinical studies.

## Figures and Tables

**Table 1:** Plasma total proteins, albumin, total globulins, and fibrinogen concentrations in reservoir bag and effluent bag at 1.5, 2, and 3 processed plasma volumes during an ex vivo session of double-filtration plasmapheresis.

PV Processed	Total Proteins (g/L)		Albumin (g/L)		Total Globulins (g/L)		Fibrinogen (g/L)	
	RB	EB	RB	EB	RB	EB	RB	EB
**Baseline**	38	-	23.2	-	14.5	-	1.3	-
**1.5 PV**	16	79	11.2	38.6	5.2	40.3	<0.4	1.1
**2 PV**	11	61	7.5	33.3	3.8	27.6	<0.4	1.5
**3 PV**	9	44	5.6	25.2	3.1	18.4	<0.4	1.0

PV, plasma volume; RB, reservoir bag; and EB, effluent bag.

**Table 2:** Plasma globulins concentration (g/L) in reservoir bag and effluent bag at baseline, 1.5, 2, and 3 processed plasma volumes, during an ex vivo session of double-filtration plasmapheresis.

PV Processed	α1-Globulin (g/L)		α2-Globulin (g/L)		β1-Globulin (g/L)		β2-Globulin (g/L)		γ-Globulin (g/L)	
	RB	EB	RB	EB	RB	EB	RB	EB	RB	EB
**Baseline**	1.7	-	4.4	-	2.8	-	3.7	-	1.9	-
**1.5 PV**	0.9	2.5	1.3	15.8	1.0	7.0	1.3	8.8	0.7	6.2
**2 PV**	0.6	2.4	0.9	10.1	1.0	4.7	0.8	6.1	0.5	4.3
**3 PV**	0.4	1.9	0.8	6.1	0.8	3.0	0.6	4.4	0.5	3.0

PV, plasma volume; RB, reservoir bag; and EB, effluent bag.

**Table 3:** The mass in grams (g) of total protein, albumin, total globulins, γ-globulins, and fibrinogen in a reservoir bag at baseline and in the effluent bag at 1.5, 2, and 3 processed plasma volumes (PV). Net percentage loss (%) of albumin, globulins, γ-globulins, and fibrinogen during the ex vivo double-filtration plasmapheresis session.

PV Processed	Total Protein (g)		Net Protein Loss (%)	Albumin (g)		Net Albumin Loss (%)	Tot Globulins (g)		Net Total Globulins Loss (%)	γ-Globulins (g)		Net γ-Globulins Loss (%)	Fibrinogen (g)		Net Fibrinogen Loss (%)
	RB	EB		RB	EB		RB	EB		RB	EB		RB	EB	
**Baseline**	63.9	-	-	39	-	-	24.4	-	-	3.2	-	-	2.2	-	-
**1.5 PV**	-	20	32	-	9.7	25	-	10	41	-	1.6	50	-	0.27	13
**2 PV**	-	25	40	-	13.7	35	-	11.3	47	-	1.8	57	-	0.61	28
**3 PV**	-	27	42	-	15.5	40	-	11.3	47	-	1.8	57	-	0.61	28

The mass in grams (g) of the plasma protein fractions at baseline was calculated as follows: [(solute concentration in reservoir bag (g/L) × total volume (L) reservoir bag)]. The mass in grams (g) of the plasma protein fractions being removed at 1.5, 2, and 3 processed plasma volumes was calculated as follows: [(solute concentration in effluent bag (g/L) × total volume (L) effluent bag)]. The net percentage loss of the different protein fractions was calculated as follows: (mass (g) of solute in the effluent bag at target PV)/(mass (g) of solute in reservoir bag at baseline)] × 100. PV, plasma volume; RB; reservoir bag; and EB, effluent bag.

## Data Availability

The data supporting the conclusions of this article will be made available by the authors on request.

## Abbreviations

The following abbreviations are used in this manuscript:

DFPP	Double-filtration plasmapheresis
PV	Plasma volumes
